# Epigenetic changes in dendritic cells in sepsis-associated immunosuppression

**DOI:** 10.1186/cc13001

**Published:** 2013-11-05

**Authors:** Raphael Molinaro, Papp Attila, Adriana Ribeiro Silva, Hugo Caire Castro-Faria-Neto, Claudia Farias Benjamim, Laszlo Nagy, Patrícia Torres Bozza

**Affiliations:** 1 Immunopharmacology Laboratory, IOC/FIOCRUZ, RJ, Brazil; 2 Nuclear Hormone Receptors Laboratory, Debrecen, Hungary; 3 Inflammation Laboratory, UFRJ, Brazil

## Background

Sepsis is a systemic inflammatory response syndrome against infection, which can develop in sepsis-associated immunosuppression. Actually, several inflammatory dysfunctions have been described in dendritic cells (DCs), which could be responsible for impairing the immune response towards the secondary infection, although how these stable modifications maintain is still unknown. Our hypothesis is that DCs from post-septic mice have chromatin alteration and differential microRNA expression.

## Materials and methods

To investigate the global gene expression, post-septic and Sham-derived BMDC were infected or not with BCG for 24 hours. Total RNA were collected and the gene expression profile was assessed by Affymetrix GeneChip technology. The gene expression profiles were classified by Gene Ontology (GEO). Also, the microRNA analysis was obtained from Affymetrix microarray. To investigate the chromatin modifications, post-septic and Sham BMDC were performed to Chip-Seq analysis.

## Results

Supervised analysis identified a set of 2,755 genes that distinguished very accurately between post-septic BMDC and Sham BMDC. The gene expression signature showed 1,805 stimulated genes and 950 inhibited genes in post-septic BMDC compared with Sham BMDC. The gene expression signature of post-septic BMDC provided a molecular and functional profile based in GEO. It is noteworthy that post-septic BMDC were mostly found in the downregulated genes to encode proteins involved in the biological pathways of the inflammatory process (IL-1α, IL-12, CD28, TLR2, Hmgb1, CCL2), lipid metabolism (FABP4, Elovl2, PTGS1, PPARδ) and histone modifications (ACAT3, CBx2, Oip5, Hist2hX). When post-septic and Sham BMDC were infected with BCG, downregulated gene sets were classified in 130 significant GEO terms (mainly involved in inflammatory and lipid metabolism process) while surprisingly upregulated gene sets were classified in 10 significant GEO terms (nine inflammatory processes of 10 terms). In microRNA expression, we observed higher microRNA expression in post-septic compared with Sham BMDC. When BMDC were infected with BCG, post-septic BMDC exhibited higher numbers of microRNA compared with Sham BMDC. Furthermore, we assessed the presence of H3K27ac and H3K4me3 in inflammatory (IL-10, TNFα, IL-6 and TGF-β) and lipid metabolism genes (ABCA1, PLIN2, CD36 and FABP4). Both H3K4me3 and H3K27ac on PLIN2, CD36 and FABP4 gene bodies were reduced and the presence of H3K4me3/H3K27ac was increased on TNFα and TGF-β gene bodies.

## Conclusions

These results demonstrate the global gene expression signature, higher microRNA expression and H3K4me3/H3K27ac profile on chromatin structure in post-septic BMDC. The present study suggests epigenetic changes may play a role in transcriptional regulation in post-septic DCs.

